# Health by Good Living

**Published:** 1870-03

**Authors:** 


### HEALTH BY GOOD LIVING.

THIS is the title of a book just published by Hurd & Houghton, of New York, No. 13 Astor Place. An extract from the book will give a better idea of it than a review, and as it treats of a class of ailments found more or less in every household, and as the unmedicinal treatment of them has effected a permanent cure in numerous cases, it is believed that the volume ought to secure very general attention. The ailments more especially considered are, biliousness, dyspepsia, neuralgia, and nervousness; the meaning of these terms is plainly defined, their causes enumerated, their mode of action explained, and the treatment marked out with such precision and definiteness, that mistakes and misapprehensions are impossible; and more, the plan of treatment is available to all to a successful result without any other expense than that of the book itself; the author rides no hobby; advises no medicine; recommends no patent or machine to grind out cures.

Other subjects are necessarily introduced and discussed which have an incidental connection with the main topic, as will be seen by the following extracts:—

"WHITE AND BROWN BREAD.

| Vegetable Food. | Pounds per Bushel. | Amount of Nitrogen. | Mineral matter. |
|---|---|---|---|
| Fine flour. | 56 | 1 70 | 0 71 |
| Seconds. | 56 | 1 86 | 0 99 |
| Sharps. | 26 | 2 40 | 2 90 |
| Fine Pollards. | 16 | 2 43 | 6 00 |
| Bran. | 12 | 2 40 | 7 00 |

"Much has been said of the superior healthfulness of brown bread over white, and still the masses will use the whitest flour they can get. The writer knows a very wealthy owner of flour-mills, who, from principle, uses bran-bread on his family table, but supplies the whitest flour to his servants, otherwise they would not live with him a week. Is the instinct of the servant nearer right than the intellect of the master?

"From the above table it will be seen that a bushel of bran has nearly seven times as much mineral matter as a bushel of fine flour. It is this mineral matter which mainly gives strength to the bones, and beauty and lastingness to the teeth, and vigor to the brain, and power to the muscles; that is, this mineral matter, or 'ash,' utilizes the nitrogen and the carbon derived from our food. But investigation shows that half of the bran is indigestible, even if passed through half-a-dozen animals in succession; secondly, it is so irritating, by its jagged points coming in contact with the delicate coating of the bowels, that it forces the food through the alimentary canal in healthy persons before it is fully digested, hence causes waste. Hence, as was said in a previous page, it is beneficial to those whose bowels are too slow, constipated; hence the caution was there given, that when taken to remove constipation, it should be discontinued when the desired result was secured; thus having something to fall back upon in case of further need in that direction.

"Hence, working and observant men seem instinctively to have chosen the whitest bread, as more easily digested, and as giving more strength to work. A middle course would seem, in the present state of our knowledge, to be the most desirable, neither to use the finest flour, nor the whole product of the grain known as seconds flour, which should contain eighty per cent. of the whole grain. It has been said that the very outer skin has been removed, thus yielding eighty-eight per cent. of the grain, excluding only the perfectly indigestible portion.

"GOOD BREAD.

"One other reliable fact may here be stated in reference to wheat bread, as it is on every table. One hundred pounds of flour will make one hundred and thirty-three and one-third pounds of bread; that is, out of three hundred pounds of flour, our baker sells us four hundred pounds of bread. But he is not satisfied until he adds one-third to that profit, by either putting in some alum, which, while it whitens the loaf, makes it capable of holding one-third more water. Or, if three or four pounds of rice are boiled three hours in three gallons of water, and this is mixed with the flour in the dough, a large increase of weight is added to the bread. To make good bread, thirty-seven per cent. of water should be added to the flour; that is, sixty-three pounds of flour, and thirty-seven of water.

"MILK.

"Perfect food is prepared for the young of animals and man; hence in milk and the egg are found all the elements necessary for growth and support. In ten pounds of milk there are of

| | |
|---|---|
| Water | $8\frac{5}{10}$ pounds. |
| Caseine or cheese | $\frac{5}{10}$ pound. |
| Sugar | $\frac{4}{10}$ pound. |
| Butter | $\frac{3}{10}$ pound. |
| Lime, etc | $\frac{3}{10}$ pound. |

Goats' milk, 80 parts caseine, 40 sugar, 40 butter.
Cows' milk, 63 parts caseine, 28 sugar, 40 butter.
Human milk, 32 parts caseine, 26 sugar, 29 butter.

"Butter and sugar warm the system; the caseine, representing the cheesy portion of milk, supplies strength and repairs the waste; hence the young of animals, being obliged to use their limbs so much earlier than children, they must have more caseine to repair the greater waste made by the necessity of a greater amount of effort needed for their out-door life and various necessities peculiar to their state and condition.

"In the following table is given the proportion of the nutriment and the proportion of fuel in a given quantity of food:—

Milk contains one proportion of nutriment, 2 of fuel.
Beans contain one proportion of nutriment, 2½ of fuel.
Oatmeal contains one proportion of nutriment, 5 of fuel.
Barley contains one proportion of nutriment, 7 of fuel.
Wheat contains one proportion of nutriment, 8 of fuel.
Potatoes contain one proportion of nutriment, 9 of fuel.
Rice contains one proportion of nutriment, 10 of fuel.
Arrowroot contains one proportion of nutriment, 26 of fuel.
Tapioca contains one proportion of nutriment, 26 of fuel.
Sago contains one proportion of nutriment, 26 of fuel.
Starch contains one proportion of nutriment, 40 of fuel.

"The last-named articles are given to young children, because they require a great deal of warmth. But they need more than warmth; if fed on these alone, they would soon die; hence milk must be added to these, as it contains materials for growth and repair.

"If human milk be considered as having 100 of nutritive equivalents,—

| VEGETABLES DRIED. | |
|---|---|
| Rice will have | 81 |
| Potatoes | 84 |
| Maize | 100 |
| Rye | 106 |
| Radish | 106 |
| Wheat | 119 |
| Barley | 125 |
| Oats | 138 |
| White bread | 142 |
| Black bread | 166 |
| Peas | 239 |
| Lentils | 276 |
| Haricots | 283 |
| Beans | 320 |

| ANIMAL FOOD. | |
|---|---|
| Human milk | 100 |
| Cows' milk | 237 |
| Yelk of eggs | 305 |
| Oysters | 305 |
| Cheese | 331 |
| Eel | 434 |
| Muscle | 528 |
| Beef liver | 570 |
| Pigeon | 756 |
| Mutton | 773 |

"A nation, as well as the individual, should know how to use its food economically. This table is suggestive in that direction; and it will interest the reader to compare the amount of nutriment contained in the different articles above named, making human milk the starting-point; thus a pound of mutton contains nearly as much nourishment as eight pounds of milk. The muscular strength of a nation depends upon the proper use and proportions of the various kinds of food eaten; and it has been well said that the political influence of a nation is as much dependent upon the muscular strength of the people as upon their intelligence and commercial activity. Englishmen and roast beef are synonyms; and for centuries past the English nation has been the most powerful, the most influential nation on the globe; a long-lived, intellectual, and powerful race, as to the individuals composing it, founded on vigorous 'health' as a result of 'good living.' The preceding table is only to be used for comparative purposes, as approximative, because other practical considerations would modify the result in any given case. But a table has been prepared by Dr. Letheby, one of the most eminent men in the medical profession in Great Britain, and was communicated to the Society of Arts in one of the Cantor Lectures, and may be considered as authentic. To prepare such a table has required an immense amount of labor and research, it will be of permanent scientific and practical value, and reflects great credit on the indefatigable investigator.

"HOW MUCH TO EAT.

"Eminent physiological investigators have found that the amount of food daily necessary to keep a person in health must be enough to yield four thousand one hundred grains of carbon, and one hundred and ninety grains of nitrogen; counting seven thousand grains avoirdupois to one pound, it is possible for a person to live in health on an amount of food which would yield a little over half a pound of nutriment, not counting the water drank, or the air breathed.

"Dr. Edward Smith, of London, says that the amounts required for sustaining health, are for an

| | Grains of Carbon. | Grains of Nitrogen. |
|---|---|---|
| Adult woman, | 3,900 | 180 |
| Adult man, | 4,300 | 200 |
| Average adult, | 4,000 | 190 |

"These proportions would be obtained in about two and a quarter pounds of good wheat bread. On this foundation the following interesting table has been prepared.

"OBJECT OF EATING.

"We eat to live; and if we eat wisely of what he has provided who giveth us all things richly to enjoy, we shall live well, healthfully, and long.

"To eat wisely, we must adapt our food to our age, to the various occupations and callings of life, and to the temperaments of the system. This may appear to be a very discouraging complexity in the very outset, but it is only seeming, for Infinite wisdom and Fatherly beneficence has implanted within us a kind of self-acting guide, has made it a part of our being, and, if it is wisely deferred to and considerately followed, half the ordinary diseases of humanity would be blotted out, and a score of years added to the average duration of civilized life.

"As soon as the little duck breaks its shell, it waddles toward the water, and sails away over the bosom of the tiny pond right gracefully.

"The humble climbing vine will direct its course straight to the nearest bean-pole; and the roots of flower, and shrub, and tree, as they delve down into the hard earth, will ferret out the richness and the moisture of the soil, taking the very shortest course to the more favored spots; and so the infant, in the first hours of its existence, greedily partakes of its mother's milk, which contains in large proportions the elements which supply the first necessities of infantile existence. This wise and friendly guide to animal and plant and man is called 'Instinct,' and is our kindly mentor and preserver from the first cry of infancy until the fiat of the Maker calls the patriarch home to his bosom in heaven! This instinct is chiefly our guide during the undeveloped mental condition of infancy and childhood; at those accidental seasons of later life when reason may be in abeyance from disease or other causes; and when action is necessary to the preservation of the body in the various emergencies to which humanity is subject, and which action must be taken before reason has had time to assert herself. This is familiarly illustrated in every-day life, when the child, or even the man, stumbles and falls forward, throwing his hands before him, to preserve the more important part, the face, from disfigurement. The writer has seen men fall from the tops of houses, and from a mile in height, and the body was always noticed to assume the shape of a ball, as if to present the smallest possible surface for the terrible contact. The cattle in the field, in cold, windy, rainy weather, and in the sleets of winter, draw up the body or contract the back to a circular form, thus presenting less surface to the chilling blast. Men, in very cold winds, thus contract themselves in walking or in sitting down, in the out-doors, by which means less of the surface of the body is exposed to the wind, and, as a consequence, less heat is carried from the system. This is the work of Instinct; but few, comparatively, know the philosophy of it until explained to them, as now.

"It is this same Instinct, exhibited in another direction, which calls for food to sustain nature. The animal creation is probably guided by it altogether in eating, as to time, quality, and quantity, and, as we see, is in a measure exempt from disease, dying more by age and violence than by sickness.

"The animal, the infant, and the demented, seem to be guided in their eating, mainly at least, by the instinct of a natural appetite; and if men were more under this same influence, and were less the slaves of appetites which are artificial and acquired, it will scarcely be denied that they would be largely exempt from many of the maladies which now afflict civilized society.

"WE EAT TO GROW.

"The blade of grass of to-day is taller than it was yesterday; the sapling larger than a year ago; and the huge oak stretches its giant arms higher in the air and wider than a century since. All these grow in part by feeding on the air, by drawing its component elements to themselves, then condensing them into more solid substances, which are incorporated with themselves, and thus become part and parcel of the living body; but, at the same time, the little roots below stretch out their tiny tendrils deep and broad, and, by a mysterious agency, dissolve the solid earth, and its more solid metals, into fluids and thinner gases, and then, drawing them up into the growing body, they become solidified, and make a part of the living whole. In ways like these atom by atom is added to blade, and stalk, and towering tree, and thus do they grow, day by day. Hence, vegetation eats to grow, by appropriating exterior unliving things to its own living uses. It takes the inanimate earth and air, and makes them a living part of its living self, and in turn is appropriated to the sustentation of a form of life as much higher than itself as it was above the baser dust on which it fed; for upon this lower life of grass, and herb, and tree, the cattle on a thousand hills are fed, and they at last are given to men to eat, and thus become incorporated in turn into a still higher existence; are made a part and parcel of the living embodiment of man; a candidate for an immortal state beyond, at so infinite a remove above the beasts which perish that he is even now but a little lower than the angels, and is ordained to have a nobler name, a higher place, and a grander destiny than they!

"WE EAT FOR REPAIR.

"In a deep, damp, dark dungeon, writes a lady of world-wide renown, I saw him chained to the cold, slimy, stone floor. His largest liberty was three steps back and forth. That ugly, clanking chain never by any possibility allowed him to go further; and all day long, and sometimes all night, for so many mournful years, the weary, naked foot had fallen on the same hard spot on that pitiless stone, and had worn into it a deep hollow; yes, the solid stone had worn away, but not the soft skin of that unhappy prisoner's foot. It gave no sign of wearing out. The stone was dead matter, and when a portion of it, ever so infinitesimally small, was worn away, there was no power to replace it. The sole of the foot was a living thing. It wore away faster, much faster, than the more solid stone; but as soon as one particle was displaced another was deposited in its stead; as when a soldier falls in the front rank of battle his brave comrade from the rear takes his position, and the line remains always full. This particle supplied to the worn foot is brought to it in the blood, which circulates to every pin-point of the body, but that particle is supplied from the food eaten. Hence, we not only eat for purposes of warmth and growth, but we eat for repair.

"All machinery, the most perfect piece of mechanism which ever came from human hands, will wear out, because there is friction. Its cogs, its wheels, its bearings, its axles, and its cylinders all move upon one another, more or less directly. Such motion implies friction, and friction causes loss of substance necessarily. Millions of money are expended every year for the purchase of oils and other lubricants, to lessen the tremendous wear and waste in the running of our locomotives, the trains on our railroads, and the machinery of our numberless mills and manufactories. But the living human body came from the hands of the Infinite One. It is the perfection of mechanism, and has within itself the power of growth and development; and more, it makes its own repairs and provides its own lubricants; works incessantly, day and night, summer and winter, seed-time and harvest, for a hundred years. It never stops, it never wears out, until the work is completed for which it was made, and the Master-builder bids it run no more! It is made of its hundreds of muscles, and bands, and sockets, and hinges, and pulleys, all playing upon or dragging across each other. The very smallest of these motions involves waste; indeed, not a single crook of the finger, not a bend of the arm, not a twinkle of the eye, not a thought of the brain, but is at the expense of some solid portion of the human machine; and yet, at the end of a century, it remains a whole in all its parts, while the most perfect constructions of man come to a dead stop in a very few months, and would stand still forever, unless some new cog, or pin, or pulley was supplied. But the tongue which speaks to-day spoke a hundred years ago just as well, and the eyelid winks as easily at fourscore as in infancy. It does not even wink tiredly. And all this, not because there are no wastes of substance in this wonderful frame of ours, but because they are as promptly repaired as made.

"EATING DOWN-TOWN.

"Men do not dine down-town long, before they get into the habit of 'taking something' at their meals. In fact, most of the eating-houses calculate to make as much in the way of profit on what their customers drink, as on what they eat; and boys, and clerks, and young men, very soon begin to feel that it looks manly to call for something at lunch. They think it adds to their importance in the estimation of the waiters to take a glass of wine, or beer, or other drink ; just as a little earlier they thought it ' manly ' to smoke a cigar, or ' take a chew.' Men often invite their friends to go and take a lunch with them, when it is expected, as a matter of course, some form of stimulant will be ordered ; this is, sooner or later, reciprocated ; and thus, the man who, a while ago, had taken a glass only occasionally, finds himself taking it every day ; and if from any cause he does not get it, there is a disagreeable sensation of wanting something, and this is not appeased until the accustomed glass is supplied ; and this is the beginning of the end, the miserable end, of filling a drunkard's grave, leaving a ruined estate, a broken-hearted wife and children in want, in destitution, and in desperation, and too often soon ready, in their recklessness, to do and dare any thing. Very many cases have occurred in New York City, of gentlemen who once would have been shocked to have had a brandy bottle beside them at their own table, in the presence of wife and children, yet in a very short time have gradually fallen into the habit, at down-town lunch, of having a glass of ale, or beer, or wine, ending in clear brandy.

"LIQUOR AND LUNCH, OR WALL STREET SENSIBILITY.

" It is said that Wall Street is the most sensitive spot on the globe. That is, it sees, on the instant, what effect national occurrences, at home or abroad, tend to have on monetary affairs ; so alive are men, and so acute, in seeing what is to their own interest. Capital is careful ; and it may surprise the reader very much to know that the practice of drinking liquor in connection with lunch has become so general in New York with young men, and clerks, and others in subordinate positions, and the ill-effects are so apparent, that quite a number of the largest banks in and about Wall Street have for more than a year been in the habit of having substantial lunches spread in their own buildings, under the very eyes of the more responsible bank officers, so that their business may not suffer from their clerks indulging in liquor with their lunch. And if moneyed men, for pecuniary considerations, expend large amounts every year to guard against the evils referred to, it is very certain that the necessity for it has been forced upon their attention by incontrovertible facts. And it is high time for parents and guardians, and even sisters and wives, to consider whether they have not a more than pecuniary interest in devising measures to counteract the mischief of dining down-town as to the male members of their households."

### SAVE YOUR DAUGHTERS.

Several pages are devoted to the care of our daughters. It is stated that a large proportion of cases of dyspepsia and consumption have their foundations laid when children are in their teens, arising from habits of eating at home, especially girls who are all the time about the house and get into the habit of nibbling at whatever eatable happens to catch their eye; and why and how this causes dyspepsia or general ill-health.

### FEMALE BOARDING-SCHOOLS,

As a class, are strongly spoken against, as places where mind and morals and physical constitution are often irreparably injured; bad habits are fostered; wrong ambitions; the affections become weaned from home and home influences; that to grow up affectionately, loving, and healthful and pure, a mother's care, and a mother's loves and caresses are forever needed, and for these a hired substitute is a miserable mistake.

### CHILDREN'S EATING.

Several pages are devoted to this subject. Children should be allowed to eat in unrestraint in their natural mirthfulness; thus will they eat slowly, and digestion be promoted by that hilarity which all acknowledge to be an important table accompaniment. The cruelty of keeping the little ones always in a straight jacket is thus illustrated.

### A SWEET LITTLE GIRL

"Was allowed to be taken by the servant to visit a lady whom the little thing was proud and glad to see. The mother knew that the child would be presented with something nice to eat, but parted from her darling with this injunction: 'Be sure, my little pet, not to do any thing that is not proper.' The little child was brought into the parlor and placed on a chair, while the nurse retired outside into the hall. In due time the young visitor was supplied with a delightful apple, healthful, soft, and juicy, which was eaten with great relish. After a considerable time spent in silence, restlessness, and apparent deliberation, the little thing was noticed to be making a desperate effort at some deliverance. At last it came thus wise: 'Aunty Harper, do you think it would be proper for me to ask you for another apple?'

"To hold such tyrannies over children in connection with their eating, is against all reason and common sense. Let them alone in their eating; leave them to their instincts, to their natural mirth, and joyousness, and harmless chattings; these will prove a more effectual preventive of fast eating than all the parental injunctions of a young life-time. Children should be allowed to eat with children, with their equals, at least in unrestraint; for it prevents excesses, it promotes digestion, insures health, and makes of eating what a wise and kind Providence intended it should be, the means of life and a daily source of enjoyment, pleasure, and happiness.

"Let your children alone when they gather around the family table; it is a cruelty to hamper them with manifold rules and regulations about this, and that, and the other. As long as their conduct is harmless as to others, encourage them in their cheeriness. If they do smack their lips, and their suppings of milk and other drinks can be heard across the street, it does not hurt the street: let them alone. What if they do take their soup with the wrong end of the fork? it is all the same to the fork: let them alone.

"Suppose a child does not sit as straight as a ramrod at the table; suppose a cup or tumbler slips through its little fingers and deluges the plate of food below, and the goblet is smashed, and the table-cloth is ruined: do not look a thousand scowls and thunders, and scare the poor thing to the balance of its death, for it was scared half to death before; it 'didn't go to do it.' Did you never let a glass slip through your fingers since you were grown? Instead of sending the child away from the table in anger, if not even with a threat, for this or any other little nothing, be as generous as you would to an equal or superior guest, to whom you would say, with a more or less obsequious smile, 'It's of no possible consequence.' That would be the form of expression even to a stranger guest, and yet to your own child you remorselessly, and revengefully, and angrily mete out a swift punishment, which for the time almost breaks its little heart, and belittles you amazingly. The proper and more efficient and more Christian method of meeting the mishaps and delinquencies and improprieties of your children at the table is either to take no notice of them at the time, or to go further, and divert attention from them at the very instant, if possible, or make a kind apology for them; but afterward, in an hour or two, or, better still, next day, draw the child's attention to the fault, if fault it was, in a friendly and loving manner; point out the impropriety in some kindly way; show where it was wrong or rude, and appeal to the child's self-respect or manliness. This is the best way to correct all family errors. Sometimes it may not succeed; sometimes harsh measures may be required; but try the deprecating or the kindly method with perfect equanimity of mind, and failure will be of rare occurrence."

The book of 277 pages can be had at all the principal book-stores in the country for a dollar and a half, and we hope it will have a tremendous sale, because we wrote it ourself and firmly believe it to be a very, very good book indeed.

